# Improving the Use of Species Distribution Models in Conservation Planning and Management under Climate Change

**DOI:** 10.1371/journal.pone.0113749

**Published:** 2014-11-24

**Authors:** Luciana L. Porfirio, Rebecca M. B. Harris, Edward C. Lefroy, Sonia Hugh, Susan F. Gould, Greg Lee, Nathaniel L. Bindoff, Brendan Mackey

**Affiliations:** 1 Fenner School of Environment and Society, College of Medicine, Biology and Environment, Australian National University, Canberra, Australian Capital Territory, Australia; 2 Antarctic Climate & Ecosystems Cooperative Research Centre, Hobart, Tasmania, Australia; 3 Centre for the Environment, University of Tasmania, Hobart, Tasmania, Australia; 4 Griffith Climate Change Response Program, Griffith University, Gold Coast, Queensland, Australia; 5 Australia Research Council, Centre of Excellence in Climate System Science, Hobart, Tasmania, Australia; 6 The Commonwealth Scientific and Industrial Research Organisation, Oceans & Atmosphere Flagship, Hobart, Tasmania, Australia; University of New England, Australia

## Abstract

Choice of variables, climate models and emissions scenarios all influence the results of species distribution models under future climatic conditions. However, an overview of applied studies suggests that the uncertainty associated with these factors is not always appropriately incorporated or even considered. We examine the effects of choice of variables, climate models and emissions scenarios can have on future species distribution models using two endangered species: one a short-lived invertebrate species (Ptunarra Brown Butterfly), and the other a long-lived paleo-endemic tree species (King Billy Pine). We show the range in projected distributions that result from different variable selection, climate models and emissions scenarios. The extent to which results are affected by these choices depends on the characteristics of the species modelled, but they all have the potential to substantially alter conclusions about the impacts of climate change. We discuss implications for conservation planning and management, and provide recommendations to conservation practitioners on variable selection and accommodating uncertainty when using future climate projections in species distribution models.

## Introduction

Species distribution models (SDMs) are one of the most important tools currently available to assess the potential impacts of climate change on species [1]. They are commonly used to project potential future changes in the geographic ranges of species [2], [3], estimate extinction rates [4], [5], examine the efficacy of existing reserve systems [6], [7] and prioritise biodiversity conservation efforts [8], [9]. However, a range of factors influence the results of SDMs under future climatic conditions, including the choice of statistical model, variable selection, climate model range and emissions scenarios [10], [11], [12].While many of these issues have been highlighted in the scientific literature (for example, [13], [14], [15], [16], [17], [18], [19], [20], [21], [22]), less attention has been given to providing explanations and practical solutions to assist conservation planners and managers apply the results of SDMs. Yet these issues contribute to cascading uncertainty that makes decisions based on modelled future projections very challenging.

The emphasis of much ecological research in this area has been the validation of statistical models [11], [23], [24], [25], and the development of ensemble approaches to represent the range in SDM outputs [10], [19], [23], [26], [27], [28], [29], [30], [31], [32], [33], [34], [35], [36]. Ensemble approaches produce multiple distribution maps that require summarising before interpretation is possible. Several methods have been suggested to achieve this and deal with the errors and uncertainties of different statistical models [37]. These include representing the central tendency with median or mean values; highlighting spatial extents within which either one or all models show suitable habitat; presenting the degree of model agreement with frequency histograms; or probabilistic forecasting when large numbers of models are used [37]. However, while ensemble-based statistical methods may be appropriate to deal with errors and uncertainties between models, they may not always be the best way to summarise projected habitat ranges in SDMs due to the influence of differences in variable selection, climate model range and emissions scenarios.

Global climate models (GCMs) and emissions scenarios represent a range of plausible futures [38], [39]. They are not intended to provide accurate predictions regarding the future state of the climate system at any given point in time, but to establish the envelope that future climate could conceivably occupy. For this reason, important information may be lost if an ensemble of GCMs are summarised inappropriately. For example, presenting a multi-model mean provides a ‘central estimate’ of the projections obtained under a particular selection of GCMs and does not consider the variability represented by different climate models and/or emissions scenarios.

In this paper, we assess the literature in relation to SDM projections in applied ecological studies and evaluate the extent to which uncertainties attributable to selection of climatic variables and GCMs are addressed. Some uncertainty is not taken into account with SDMs, the classic example is the physiological response of plants and animals to an atmosphere with elevated CO_2_ concentrations. In this paper the uncertainty from changing concentrations of CO_2_ in the atmosphere cannot be addressed and thus these results assume a constant atmospheric concentration for a given emissions scenario. Informed by the findings of our literature review, we explore the influence of the choice of climatic variables and approaches to assimilating multiple SDMs based on a range of climate models and emissions scenarios using two threatened species from Tasmania, Australia, as case studies. We conclude with recommendations on the use of SDMs for conservation planners and managers.

### Common approaches to SDM in applied ecology

To gain an overview of how SDMs are being used in applied studies of species distributions under climate change, we searched for articles in the ISI Web of Science database (http://www.webofknowledge.com) from 1982 to 2013, where the phrases ‘bioclimatic’ and ‘climate change’ occurred in the title, abstract or author's keywords. In SDM literature, it has become common practice in ecological studies to refer to climatic variables derived from spatial interpolation of long-term mean monthly data as ‘bioclimatic’ variables [40]. The aim of this overview was to provide a substantive sample of relevant literature rather than being a systematic review of all the literature and available databases. The search yielded 562 records, which we refined by selecting articles that contained the words ‘niche’ or ‘habitat’, resulting in a total of 221 publications (the list of reviewed articles can be found at: http://www.mendeley.com/groups/3315561/bioclim-review/papers/). Since we focused on applied studies, review articles were omitted. A total of 163 articles had constructed SDMs for current or future terrestrial species distributions. From each article, we analysed information relating to predictor variables used in the SDM, the taxa, the scale and location of the study region, and how future climate projections were used. Data from the review were analysed using R [41].

Most of the reviewed articles focused on Europe (∼30%), North America (∼17%), Africa (∼12%), South America (∼11%), Australia and Oceania (∼10%), with a small fraction on Asia (∼3%). Note that the sum of fractions is <100% because some articles were methodological and did not include case studies. The majority of studies (58%) were carried out at the regional scale (e.g. geographic extent ≥10000 km^2^ <continental), 14% were at the continental scale, and just over 10% of the articles reported studies at scales of either <10 km^2^ or between 1000 km^2^ to 10000 km^2^. A small proportion of articles (∼2%) carried out SDMs at the global scale. SDMs were used to study the potential impacts of climate change on the distributions of a wide range of taxa, including birds (∼43%), reptiles and amphibians (∼32%), plants (∼19%), invertebrates (∼12%) and mammals (∼10%) with some articles modelling more than one taxon.

From the reviewed literature, we identified three common approaches to variable selection. The first common approach uses all available bioclimatic variables without justification. The BIOCLIM software, a sub-package of ANUCLIM [42] provides 35 variables, while WORLDCLIM [43] provides a subset of 19 bioclimatic variables. The second common approach reduces the number of bioclimatic and biophysical covariates to account for colinearity. The third selects variables based on ecological knowledge. Typically, the third involved assuming a causal relationship between a climatic variable and a theoretical or empirically established understanding of a species' eco-physiology, life history traits, patterns of movement, reproductive cycle or habitat requirements. Often the assumed causal relationship was general, such as the relationship between growing degrees days and biological growth rates [44]. A total of 119 distinct variables were used in the reviewed articles (see Figure S1) the most common being mean annual precipitation and mean annual temperature used by 43% and 37% of the articles, respectively.

Diagnostic statistics regarding performance of SDMs were reported by 55% of the articles. The most commonly used statistics to assess the performance of SDMs were the area under the receiver operating curve (AUC), Akaike information criterion (AIC), Bayesian information criterion (BIC), Kappa and R^2^. We found three approaches were commonly used to summarise the outputs of SDMs: (1) model mean tendency; (2) model agreement; and (3) the bounding box [23].

About 10% of the reviewed papers provided justification for the selection of GCM. About 40% of the reviewed articles used two or more GCMs with one or two emissions scenarios. Only seven articles [19], [45], [46], [47], [48], [49], [50] used more than 10 GCMs and acknowledged variability in these inputs. Each of these seven articles focused on testing SDM methods, rather than applying SDMs to practical conservation problems. The review database can be found in File S2.

## Material and Methods

We used two contrasting species as case studies to examine the issues related to variable selection and GCMs that commonly arise when modelling future species distributions for applied conservation: the King Billy Pine (*Athrotaxis selaginoides)*; and the Ptunarra Brown Butterfly (*Oreixenica ptunarra*). King Billy Pine is a long-lived paleo-endemic tree that grows at altitudes of 600 m to 1100 m. In contrast, the Ptunarra Brown Butterfly is a short-lived species, which is active for three to four weeks in late February to April, at altitudes from 200 m to 1200 m. These species are endemic to Tasmania, Australia, and have been listed as endangered under the *Environment Protection and Biodiversity Conservation Act* 1999. We conducted SDM experiments to examine the range in projected distribution due to (a) the three common approaches to variable selection identified in the review and (b) the three different ways of accounting for the range in SDM results from using multiple GCMs and emissions scenarios.

### Species Distribution Models

Presence data for the species were downloaded from the online database Atlas of Living Australia (http://collections.ala.org.au/public) and databases of the state conservation agency, the Department of Primary Industries, Parks, Water and Environment (DPIPWE, https://www.naturalvaluesatlas.tas.gov.au/). There were 212 unique observations for the Ptunarra Brown Butterfly from 1949 to 2007; and 672 observations for The King Billy Pine from the period 1847 to 2005.

The MaxEnt model [51] was used to model the suitability of future climate for each species. While there are several other methods for modelling species distributions (e.g. generalised linear models, boosted regression trees, mechanistic models and ensemble techniques), we used MaxEnt because it is not computationally expensive, is widely used in applied ecological studies by government agencies and research organisations, and has been shown to perform well in comparison to several other models when there are relatively few presence records available [52]. We did not use an ensemble approach because, as noted, that issue has been extensively investigated and our focus here is on tools that are usable by conservation planners and managers who generally do not have access to the high computing capacity needed for ensemble modelling. We used R [41] to run MaxEnt, with fifteen replicate runs calculated by cross-validation using 30% of the data and default values for all other parameters (iterations = 500, convergence threshold = 0.00001, and regularization value = 1). Results are presented as the relative probability of occurrence, with suitable climate identified where this value is greater than 0.5 [53].

Three MaxEnt models, that represent the three common approaches to variable selection, were run for the two species using different sets of bioclimatic predictor variables. The first model used all available variables (see Table A1 in File S1). The second model used principal component analysis (PCA) to identify a subset of available bioclimatic variables that are not strongly correlated (R^2^<0.6). The third model used expert knowledge of the target species to identify subsets of bioclimatic variables. The available bioclimatic variables represent annual trends (e.g. mean annual temperature and precipitation), seasonal trends (e.g. annual range in temperature or precipitation), and extreme or limiting environmental factors (e.g. temperature of the coldest and warmest month, precipitation for wettest or driest quarters). Model 1 used the most commonly used set of variables, based on the literature review, comprising 35 variables provided by BIOCLIM. These BIOCLIM variables defined the baseline climate.

Model 2 used a subset of uncorrelated bioclimatic variables selected using PCA that explained more than 90% of the variance. For the Ptunarra Brown Butterfly, the variables were: temperature seasonality (coefficient of variation, hereafter cv); maximum temperature warmest period; and mean temperature driest quarter and annual precipitation. For the King Billy Pine, the variables were: annual mean temperature: temperature seasonality (cv); temperature annual range; annual precipitation; annual mean radiation; radiation seasonality (cv); annual mean moisture index; and highest period moisture index.

Model 3 used variables chosen by experts based on knowledge of the species' life cycle. For the Ptunarra Brown Butterfly the selected bioclimatic variables were: March minimum temperature; April minimum temperature; annual minimum temperature; radiation with rainfall for April; and annual rainfall (Dr Peter McQuillan and Dr Phil Bell *pers. comm.*). For the King Billy Pine the selected bioclimatic variables were: maximum temperature warmest period; minimum temperature of coldest period; mean temperature warmest quarter; mean temperature coldest quarter; annual precipitation; precipitation driest period; annual mean moisture index; lowest period moisture index; and mean moisture index lowest quarter (Dr Jennie Whinam and Ms Louise Gilfedder *pers. comm.*).

We only used bioclimatic variables rather than other physical environmental or vegetation habitat variables in the model in order to provide an indication of changing climatic suitability assuming all else remains equal. Model performance was assessed using the area under the receiver operating Curve (AUC) and the information criteria Akaike information criteria (AIC), corrected Akaike (AICc), and Bayesian information criteria (BIC) calculated using ENMTools [54]. These tests can be used as an objective measure of model performance and provide guidance where there are large differences between models. The degree to which novel climate conditions are encountered is assessed with the ‘multivariate similarity surface’ (MESS) output from MaxEnt. The MESS shows similarity between each point in future projections to conditions observed during model training. In addition, similarity between the outputs for the future projections based on Model 2 for the species was quantified using the *I* similarity statistic [54]. The *I* statistic calculates pair-wise similarity between two probability distributions obtained, in this case from MaxEnt, where values close to zero represent distributions with no geographic overlap, and values close to one represent similar distributions. We used SigDiff from the R package SDMTools [55] to compute significant differences between pairs of maps, using the distribution for the current climate as a reference [56], [57].

We consulted land managers and decision makers from the Australian Department of the Environment, the Tasmanian Department of Primary Industries, Parks, Water and Environment (DPIPWE), Natural Resource Management (NRM) groups and local government to ask how uncertainty in SDM outputs could be represented to better inform conservation management and prioritisation (see File S1 for further detail on these consultations).

### Future Climate Projections

Future climate projections were derived from six global climate models that were dynamically downscaled to a resolution of 0.1° (∼10 km) across Tasmania using a regional climate model (Harris et al. 2014). Downscaling was undertaken by the Climate Futures for Tasmania project using CSIRO's Conformal Cubic Atmospheric Model (CCAM). Details of the CCAM model can be found in [58], and the modelled projections are available through the Tasmanian Partnership for Advanced Computing (TPAC) portal (https://dl.tpac.org.au/tpacportal/).

The six global climate models (GCMs) used are available from the CMIP3 archive [59]: GFDL-CM2.0; GFDL-CM2.1; CSIRO Mk3.5; MIROC3.2 (medres); and UKMO-HadCM3. Each of the six GCMs was run under both the SRES A2 and B1 emissions scenarios to represent high and low emissions scenarios respectively. These models are a reasonable representation of the range of the precipitation and temperature projections for south-eastern Australia indicated in the full set of GCMs from the CMIP3 archive. Results from three of these GCMs are presented in Section 3.1.2 as described below, and results from the six GCMs are used in Section 3.2.

Climate data were spatially interpolated to a grid cell resolution of 0.01° (∼1 km) using the ANUCLIM 6.1 package [42]. A digital elevation model (DEM) of 0.01° (∼1 km) was used as input into ANUCLIM 6.1 [42]. The DEM was aggregated from the 30 seconds (∼30 m) NASA Shuttle Radar Topographic Mission (SRTM) DEM (http://srtm.csi.org) to 0.01° using R [41].

Grids representing the difference in T_max_, T_min_, precipitation and evaporation at the pixel level were calculated for each of the six downscaled GCMs, for each month within each of three future time slices, relative to the baselines used in ANUCLIM (1976–2005 and 1970–1995 for evaporation). Future time slices are represented by mean values associated with the 30-year periods: 2010 to 2039, 2040 to 2069 and 2070 to 2099. Monthly mean values and bioclimatic variables were then calculated for the future periods.

Monthly mean values for T_max_, T_min_, precipitation and evaporation for the baseline climate, used to project current species suitable climate, were generated using the MNTHCLIM sub-package in ANUCLIM 6.1, and the 35 bioclimatic variables based on these outputs were generated by BIOCLIM.

### Species Distribution Models for future climate

As noted above, for each of the case-study species, three SDMs were calculated for baseline (e.g. current) climate conditions using each of the three different approaches to variable selection (Models 1 to 3). We then assessed the results using statistical metrics (AIC and BIC) and feedback from our species experts. We used outputs from all six available GCMs under both CO_2_ emission scenarios (A2 and B1) to calculate future climate SDMs for the two species, that is, 12 SDMs for each species giving a total of 24.

We decided that Model 2 (variable selection based on PCA) was the most accurate. We then used the outputs from Model 2 to compare the three commonly used approaches to summarising the output from multiple SDMs based on future climate (model mean tendency, model agreement and the bounding box).

Mapped projections are presented here for those SDMs that were based on three of the GCMs (UKMO-HadCM3, GFDL-CM2.0, and MIROC3.2 (medres)) under each CO_2_ emissions scenario (A2 and B1). These three GCMs were selected because they are representative of the range in projected conditions. In Tasmania, UKMO-HadCM3 is wetter than the mean of all models considered in the CMIP3 archive, GFLD-CM2.0 is drier, and MIROC3.2 (medres) is closer to the mean of all models (see IPCC AR4 Chapter 11 supplemental figure 18, http://www.ipcc.ch/graphics/ar4-wg1/jpg/fig-11-18-sm.jpg) [60].

## Results

### Species Distribution Models

#### The effect of variable choice

**Figure 1:**
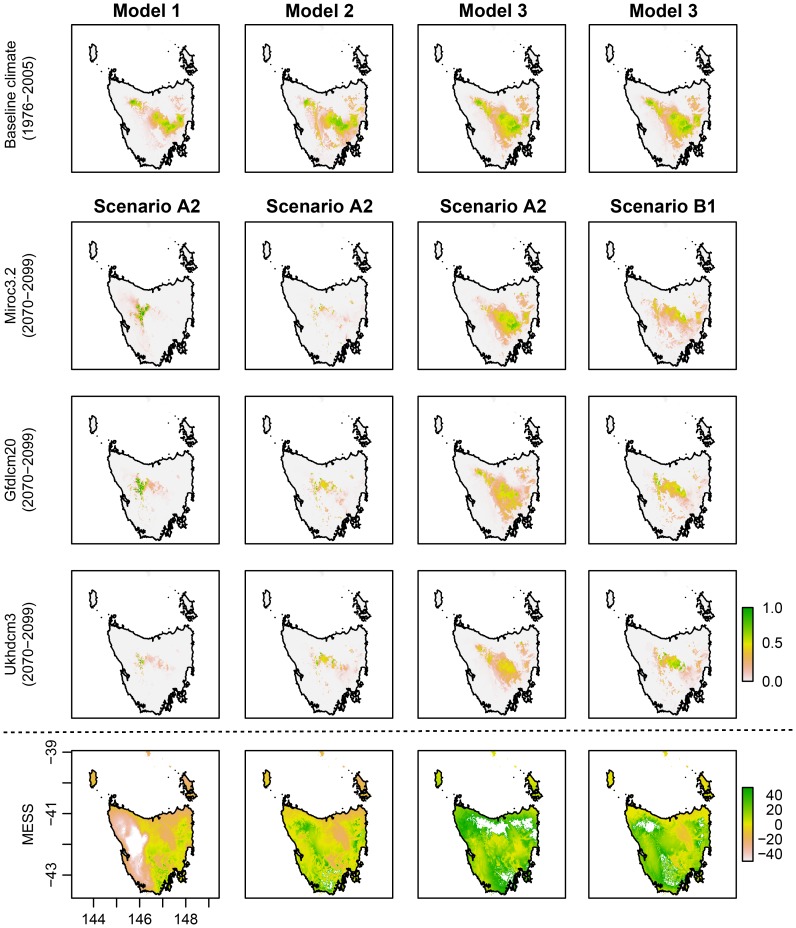
Distribution maps for the Ptunarra Brown Butterfly using baseline climate data (Row 1) and climate projections from three global circulation models (GCMs) for the period 2070–2099 (Rows 2–4) using two CO_2_ emissions scenarios: A2 for Models 1 to 3 (Columns 1–3) and B1 (Column 4, only for Model 3).

**Figure 2:**
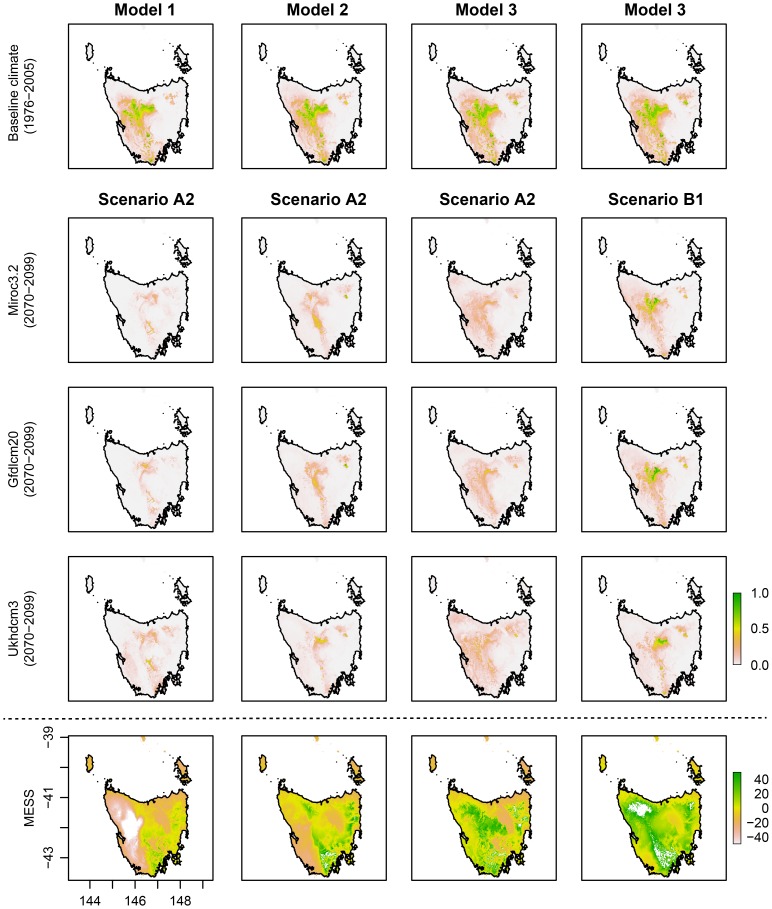
Distribution maps for King Billy Pine using baseline climate data (Row 1) and climate projections from three global circulation models (GCMs) for the period 2070–2099 (Rows 2–4) using two CO_2_ emissions scenarios: A2 for Models 1 to 3 (Columns 1–3) and B1 (Column 4, only for Model 3).

Species distribution models for the baseline period show variable choice to be an important factor in determining suitable climatic habitat for the Ptunarra Brown Butterfly (Figure 1) and less so in the case of King Billy Pine (Figure 2). However, differences between modelling approaches (Models 1 to 3) are greater under projected future climate conditions (Figures 1 and 2, and Table 1). For example, models constructed using Models 1 and 3 for the Ptunarra Brown butterfly offer sharply contrasting futures under the future climate projected by MIROC3.2 (medres). Based on Model 1, the butterfly's suitable climate envelope shifts to the west, while based on Model 3 it shrinks towards the southeast (Figure 1).

**Table 1:** Variables identified as important in determining species distribution.

	Variable Importance	
	Ptunarra Brown Butterfly	King Billy Pine
**Model 1**	Mean temperature driest quarter (33%); minimum temperature coldest period (28%); radiation warmest quarter (7.7%); radiation seasonality (7%); highest period radiation (5%); radiation coldest quarter (3%)	Mean temperature wettest quarter (22.0%); radiation coldest quarter (16.1%); lowest period moisture index (10.4%); mean temperature warmest quarter (6.3%); temperature seasonality (4%); mean moisture index lowest quarter (3.8%); radiation seasonality (3.6%)
**Model 2**	Mean temperature driest quarter (53.8%); temperature seasonality (28.3%); annual precipitation (10.5%); maximum temperature warmest period (7.4%)	Annual mean temperature (49.5%), annual mean radiation (17%); radiation seasonality (12%); annual precipitation (8.2%); temperature seasonality (4.9%); annual mean moisture index (4.8%); temperature annual range (3.3%); highest period moisture index (0.2%)
**Model 3**	April minimum temperature (46%); radiation with rainfall April (22%); March minimum temperature (16%); annual rainfall (12%); annual minimum temperature (4%)	Mean temperature warmest quarter (36.8%); mean annual precipitation (14.2%); lowest period moisture index (13.1%); mean temperature coldest quarter (12%); precipitation driest period (8.2%); annual mean moisture index (5.1%); mean moisture index lowest quarter (4.2%); minimum temperature coldest period (3.9%); maximum temperature warmest period (2.5%)

Each model was ranked by its performance based on indices shown in Table 2. For both species, Model 2 (subset of bioclimatic variables selected using PCA) performed best in terms of the AICc and BIC, and Model 1 (all bioclimatic variables) performed the best in terms of the AIC. Model 1 had higher values of AICc and BIC in both species because these indices penalise models with large numbers of variables more heavily to correct for finite sample sizes and to avoid over-fitting.

**Table 2:** Statistical model selection indices.

	Ptunarra Brown Butterfly			King Billy Pine		
	Model 1	Model 2	Model 3	Model 1	Model 2	Model 3
AIC	**3996.30**	4000.22	4142.47	**7074.82**	7174.78	7302.27
AICc	4568.30	**4078.51**	4311.20	7280.61	**7233.80**	7367.39
BIC	4474.30	**4245.25**	4472.34	7678.87	**7538.00**	7681.28
AUC	**0.97**	0.94	0.93	**0.95**	0.94	0.93

Best performance for each model shown in bold.

There was little difference between the AUC values of Models 1, 2 and 3 in King Billy Pine but for the Ptunarra Brown butterfly Model 1 (all available bioclimatic variables) had the highest AUC value (0.966) and better represented the current distribution (Figure 1).

The multivariate similarity surfaces (MESS) show areas in which one or more variables were outside the range present in the training data (based on baseline climate) when considered under projected future conditions. In the Ptunarra Brown butterfly case (Figure 1), Model 3 had no negative values, while Models 1 and 2 had substantial areas with negative values, suggesting that projections in those areas should be treated with caution. Model 3 for King Billy Pine (Figure 2) also had fewer negative values than Models 1 and 2.

### Climate model and emissions scenario effects

Climate data from distinct GCM projections of future conditions also have an effect on species distribution model results and depend on the sensitivity of the species in question (Figures 1 and 2). The SDMs based on Model 2 and the selected GCMs showed substantial reductions in area of future suitable climate relative to the present. For the Ptunarra Brown Butterfly, the SDM-estimated area corresponding to >0.5 probability of suitable climate under baseline conditions was 3823 km^2^ while suitable areas using GCM-projected future climate variables were 224 km^2^ (MIROC3.2 (medres)), 566 km^2^ (UKHadC3.1) and 613 km^2^ (GFLD-CM2.0). The difference in the future suitable spatial extent therefore differed by up to a factor of ∼3. The difference between baseline and all three future climate SDMs projections, however, is clearly greater than the difference between that arising from the three GCMs. For King Billy Pine, the corresponding areas for Model 2 were 3806 km^2^, 134 km^2^ (MIROC3.2 (medres)), 154 km^2^ (UKHadC3.1) and 150 km^2^ (GFLD-CM2.0) (see Table A2 in File S1); a comparable level of projected reduction from the baseline but with less difference between GCMs.

**Figure 3:**
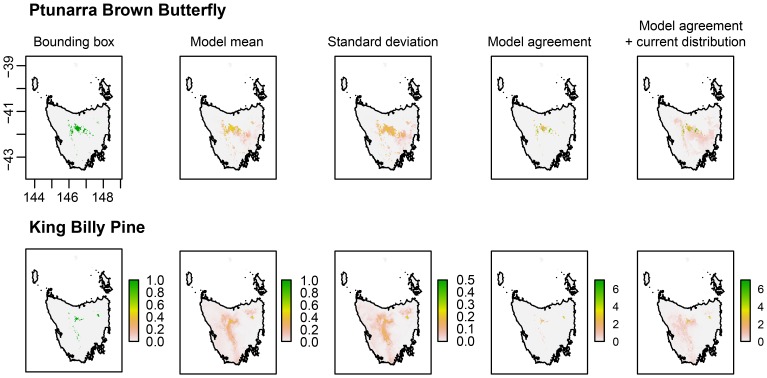
Alternative summaries for multiple species distribution models. Maps are model summaries for the Ptunarra Brown Butterfly (left) and King Billy Pine (right) using models based on six GCM inputs; means and standard deviations (Row 1); spatial extents where at least one model indicates suitable habitat (Row 2); agreement between models, where at least one model predicted suitability above 0.5 (Row 3); model agreement overlayed on the baseline distribution for the species (Row 4).

Choice of future emissions scenario also affected the results of the SDMs (Model 3 in Figures 2 and 3). The A2 emissions scenario results in reduced areas of potential suitable climate compared to the lower B1 emissions scenario and this was the case for both species (see also Figures S4 and S5). The *I* similarity statistic showed SDM predictions based on output from GCMs using A2 (higher) emissions scenarios to be substantially different from B1 in relation to baseline predicted suitable areas. Lower values of *I* found in SDMs derived from the A2 scenario represent higher degrees of difference relative to baseline SDMs (Table 3). Similarly, the difference maps present a substantial loss in suitable climate when baseline SDM are compared to the future projections for the A2 emissions scenario using Model 2 (the difference maps in Figures S2 and S3). These results, while not surprising given the strong association between emissions and rising temperature, highlight the importance of explicit recognition of emissions scenario in all SDM construction for any species with potential temperature dependence. They also point to the potential benefits to biodiversity of immediate climate change mitigation (Warren et al. 2013).

**Table 3:** The *I* similarity statistic for future projections of suitable climatic habitat for 2070–2099 using Model 2 in relation to the baseline predicted habitat for the species.

	UKHadC3.1		GFDL-CM2.1		Miroc2.0 (medres)	
	A2	B1	A2	B1	A2	B1
**Ptunarra Brown**	0.551	0.656	0.531	0.651	0.547	0.720
**King Billy Pine**	0.513	0.543	0.735	0.855	0.711	0.848

Values range from zero (no distribution overlap) to one (identical distributions). A2 values show reduced similarity in every instance.

### The effect of different approaches to summarising multiple species distribution models

Maps showing the spatial effect of the three approaches for summarising multiple SDMs are shown in Figure 3. Of particular interest is the model agreement map. While the total geographic area of suitable climate is comparable between the different future projections, they are not geographically coincident.

## Discussion

Common approaches to modelling future species distributions in applied ecological studies do not always represent the uncertainty associated with variable selection and the influence of the range in future climate projections and emissions scenarios. Using two species as case studies, we have shown that these uncertainties can affect the results of SDMs.

Ignoring the uncertainty associated with modelling future distributions may lead to over-confidence in single maps of future distributions, with consequences for conservation planning. The literature review revealed that both choice of variables and representation of future climate, by using a range of GCMs, are frequently opportunistic for SDMs in applied ecological studies. Using all available bioclimatic variables, instead of a tailored selection, increases the possibility of over-fitting species distribution models, and may lead to an over-estimate of range reduction and the likelihood of a forecasted extinction under climate change [16].

The importance of variable selection in determining the output of SDM depends on the characteristics of the species modelled. For example, variable selection may be less important for long-lived sedentary plant species such as the King Billy Pine but can be very important for mobile species with specific biological characteristics or requirements, such as the Ptunarra Brown Butterfly. For the butterfly, inclusion of different variables in the model led to widely divergent projected futures for end-of-century, ranging from close to no locations with a suitable climate [61] to maintenance of the current range.

Biological knowledge of the species can help in choosing variables amenable to ecological interpretation. Ideally, modellers, species experts and conservation practitioners should work as a team to build the SDM, interpret results and consider conservation management responses. However, such interdisciplinary exercises are uncommon and often little is known of the species' biology and ecology as they relate to climatic factors. In these cases, a range of statistical diagnostics can provide some guidance as to relative model performance, but there is no objective method for identifying the ‘best’ SDM when projecting into the future. Statistical methods of model selection alone are not sufficient to accept or reject a model, as different performance indices (e.g. AUC, AIC, BIC) can give conflicting rankings [62]. Furthermore, statistical models are a measure of internal model performance, not a measure of ecological validity.

The accuracy with which the current distribution is represented by a SDM is an important consideration. However, an accurate fit with current distributions does not guarantee that the prediction of future potential distribution will be accurate. The underlying statistical relationship between the climatic variables and the distribution of the species may change due to, for example, nonlinear functional responses of the species or indirect impacts such as an altered fire regime. Considering the extent to which a SDM is attempting to project into novel climatic conditions is also an important consideration in model evaluation. Predicted potential distribution maps with large areas in which one or more climate variables were outside the range present in the data used to train the SDM should be treated with caution.

In contrast to variable selection, the range in outputs from climate models and emissions scenarios are not species dependent and will always need to be documented when modelling future distributions. We caution against using an ensemble mean of projected climate variables as input into an SDM, for several reasons. First, each global climate model represents one plausible future among many. The Coupled Model Intercomparison Project Phase 5 (CMIP5) now includes more than 50 climate models. Although there are several quantitative model ‘skill scores’ that can be calculated for particular variables and features of the models, different models tend to perform well on some metrics and poorly on others. As a result, the IPCC avoids ranking models, and treats each equally (IPCC 2007, 2013). While multi-model GCM ensembles at large spatial scales have been shown to agree better with observations of present-day climate than single models for a range of variables [63], [64], this is not always the case at smaller scales, including the continental scale [65]. Considering multiple GCMs is therefore essential to capture the range of plausible projections. Further, the ensemble mean may not coincide with any of the climate model's projections but create a novel future climate that is merely a statistical derivative. And finally, using a multi-model GCM ensemble may not be the best approach for risk assessment in conservation planning, where considering the ‘best’ and ‘worst’ case scenarios may be a more useful framework in which to make decisions [61], [66].

Multiple SDMs need to be run using a range of climate models and emissions scenarios as input. This approach produces a range of species distribution maps (12 for each species in our case study) which need to be assessed and combined in a useful way to help inform decision-making. The method used to combine the maps (e.g. taking the mean, the median, the bounding box or the minimum overlap of the different models) will influence interpretation of the results. This can have consequences where conservation effort is deployed across the landscape since both extent and location of projected suitable climate will differ between GCMs. Over-confidence in a single map risks the possibility that conservation investments are applied to areas that do not support the target species under future climate. Ongoing monitoring is essential to track changes as the species responds to changing climatic conditions. Using a map of an ensemble mean produces a future that was not projected by any single model. Adding all areas projected to be suitable (the bounding box) will be the most optimistic outlook, while only concentrating on areas where all models agree will provide the smallest estimate of future suitability.

The managers we consulted found that the most informative summary was the map showing the agreement between the models (pixels where all models agree a potential suitability above a threshold of 0.5) in addition to the current distribution as projected by the SDM that used baseline climate data. This was considered a low-risk option to highlight priority areas for biodiversity conservation in areas that currently support a target species or community.

**Figure 4:**
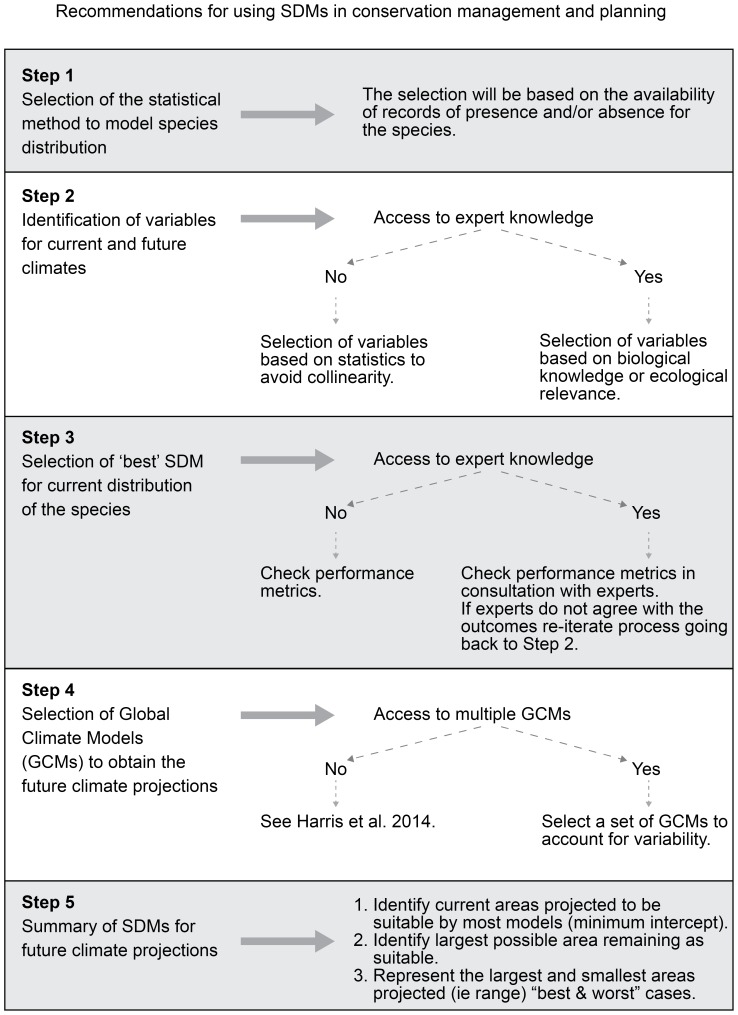
Decision tree to guide the application of SDMs under future climate to conservation management.

One aspect of uncertainty in modelling future species distribution not illustrated here is that associated with the choice of SDM. Different statistical models have been shown to have a large influence on the results of SDMs and an ensemble approach is increasingly being used to account for the range in statistical models [10], [37]. However, the main purpose of this paper was to illustrate the effect that choice of variable, climate model and emissions scenario can have on the results of SDMs for future climatic conditions. We propose a protocol for conservation planners and managers that summarises our recommendations for using SDM under future climate projections in applied ecological studies (Figure 4).

Our study highlighted that the relative change in the projected suitable climate envelope for the species is an important factor to consider. Our SDMs, based on climate variables, show that projections for the King Billy Pine are more consistent, and more consistently under threat, when viewed as relative change. A general hypothesis based on our results is that species with strong temperature dependencies are more strongly influenced by choice of emissions scenarios. If the future climate projections of the GCMs diverge, so will the SDMs, while if the climatic factor were not influential in the baseline model, the effect may be inconsequential. However, there is a threshold below which relative change is not important because, for example, the projected areas may not be of sufficient extent to support the species in any case. It is also important to note that our models, and SDM models generally, do not account for any of the other major threat to species such as habitat loss. All of these points emphasise that correct interpretation of a SDM outcome requires rigorous understanding of the inputs used in its development.

## Conclusions

Species distribution models are useful tools for, among other things, informing the conservation management of wildlife and their habitats under a rapidly changing climate. They can provide decision makers with information about the likely degree of change in a species climatic domain and geographic distribution. However, the uncertainty associated with their estimation needs to be documented and understood if their output is to be effectively applied. Systematic conservation planning can draw on this information, together with ongoing field monitoring of target species to track changes as they occur, providing the foundation for adaptive management in response to emerging circumstances.

## Supporting Information

Figure S1 The proportion of variables used in the revised literature.
(TIF)

Figure S2 Distribution maps of significant difference (SD) between the current prediction of suitable habitat for Ptunarra Brown (PB) based on baseline climate, and the future projections, based on 30 Model 2 (M2, PCA selection of bioclimatic variables). The difference maps showed when the distribution predicted significantly more (+ve) or less suitable habitat (−ve) (SD≥0.975 or SD≤0.025, respectively) and where there was no significant (ns) difference between models.
(TIF)

Figure S3 Distribution maps of significant difference (SD) between the current prediction of suitable habitat for King Billy Pine (KB) based on baseline climate, and the future projections based on Model 2 (M2, PCA selection of bioclimatic variables). The difference maps showed when the distribution predicted significantly more (+ve) or less suitable habitat (−ve) (SD≥0.975 or SD≤0.025, respectively) and where there was no significant (ns) difference between models.
(TIF)

Figure S4 Differences in annual mean temperature and annual rain within the current distribution of the Ptunarra Brwon Butterfly. Where the box spans the interquartile range, the segment inside the box 45 shows the median and whiskers above and below the box show the locations of the minimum and maximum values; the circles represent outliers.
(TIF)

Figure S5 Differences in annual mean temperature and annual rain within the current distribution of the King Billy Pine. Where the box spans the interquartile range, the segment inside the box shows the median and whiskers above and below the box show the locations of the minimum and maximum values; the circles represent outliers.
(TIF)

File S1 Table A1, List of bioclimatic variables used in the SDM for Orexeinica ptunarra and Athrotaxis selaginoides. Subset of variable using a PCA is denoted by ‘PCA’ and the subset selection made by experts is denoted by ‘x’. The Orexeinica ptunarra's experts selected monthly variables. Table A2, The area predicted to be climatically suitable for the species based on the current and future climate projections. Pixels for the Ptunarra Brown Butterfly and the King Billy Pine that with a value0.5 or more were considered as ‘suitable’. The areas are shown in km2 for Models 1 to 3 based on the SRES scenario A2, and only for Model 3 based on SRES scenario B1. Maps were projected to 25 World Geodetic System 1984, Universal Transverse Mercator coordinate system Zone 55 South.
(PDF)

File S2 (ZIP)
