# Supplementary figures and images for: Improving the Use of Species Distribution Models in Conservation Planning and Management under Climate Change
Source: PLoS One. 2014 Nov 24;9(11):e113749. doi: 10.1371/journal.pone.0113749 (PMC4242662; doi:10.1371/journal.pone.0113749)

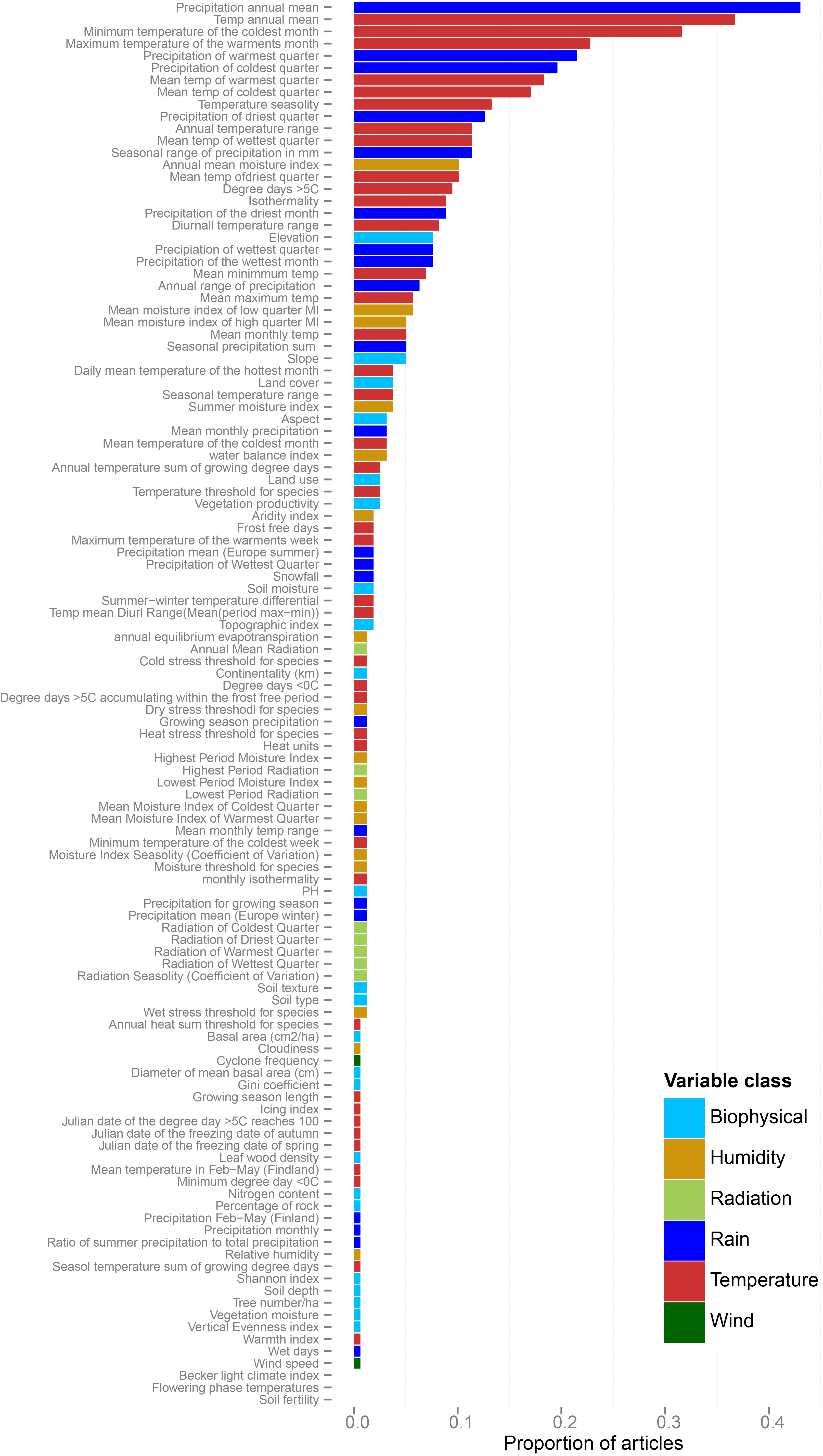

Supplement: Figure S1 — The proportion of variables used in the revised literature. (TIF) [file pone.0113749.s001.tif]

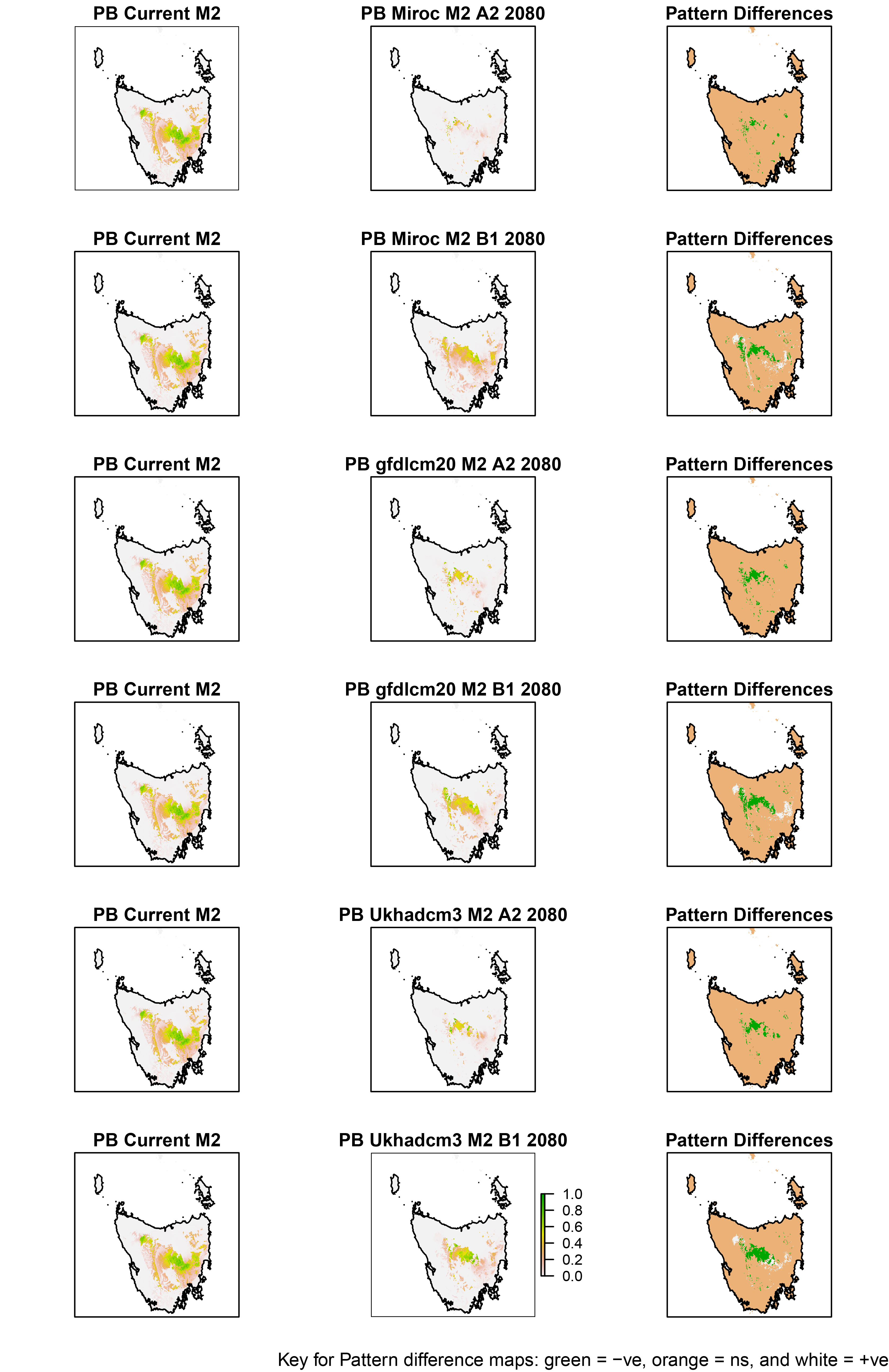

Supplement: Figure S2 — Distribution maps of significant difference (SD) between the current prediction of suitable habitat for Ptunarra Brown (PB) based on baseline climate, and the future projections, based on 30 Model 2 (M2, PCA selection of bioclimatic variables). The difference maps showed when the distribution predicted significantly more (+ve) or less suitable habitat (−ve) (SD≥0.975 or SD≤0.025, respectively) and where there was no significant (ns) difference between models. (TIF) [file pone.0113749.s002.tif]

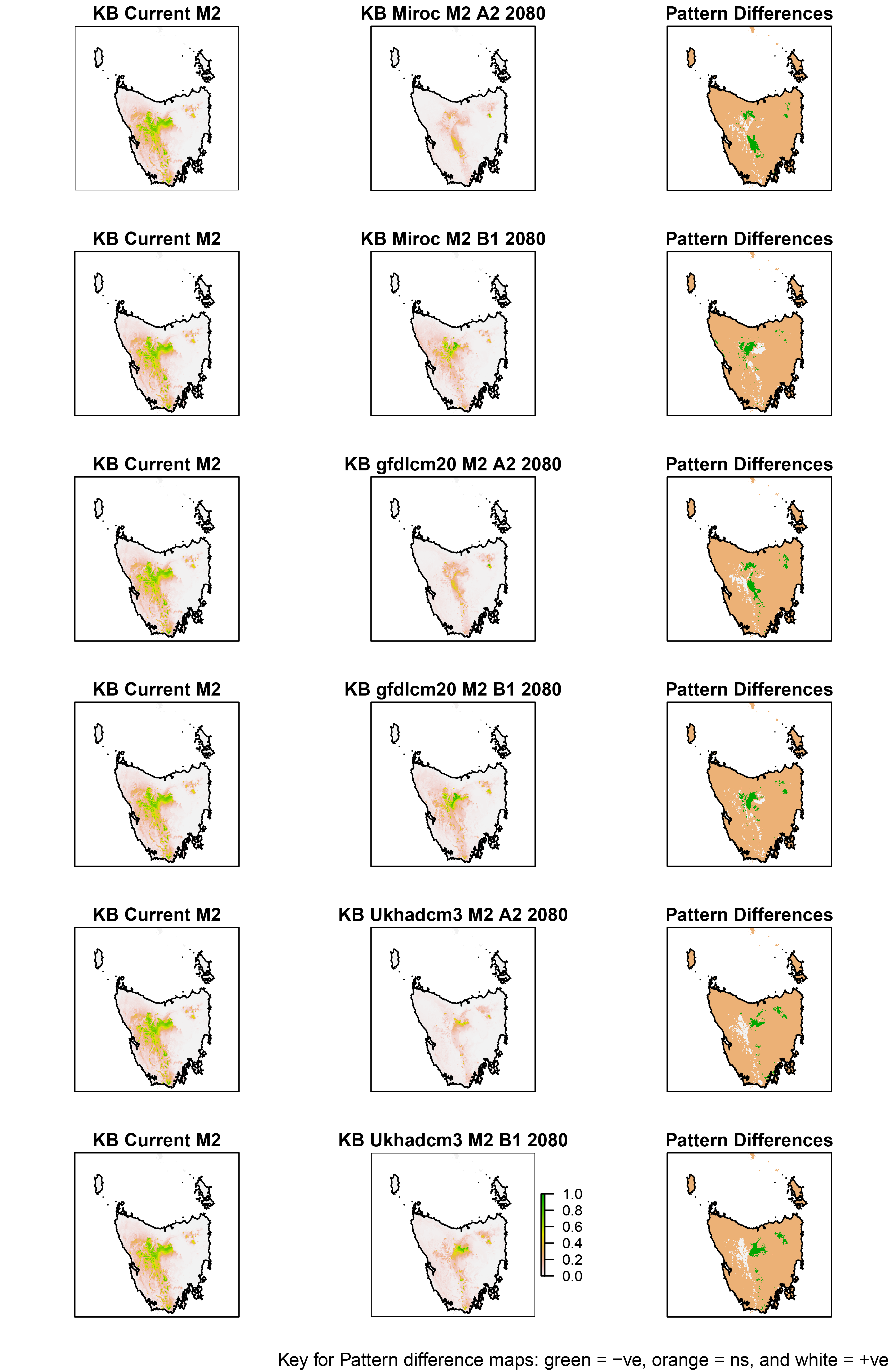

Supplement: Figure S3 — Distribution maps of significant difference (SD) between the current prediction of suitable habitat for King Billy Pine (KB) based on baseline climate, and the future projections based on Model 2 (M2, PCA selection of bioclimatic variables). The difference maps showed when the distribution predicted significantly more (+ve) or less suitable habitat (−ve) (SD≥0.975 or SD≤0.025, respectively) and where there was no significant (ns) difference between models. (TIF) [file pone.0113749.s003.tif]

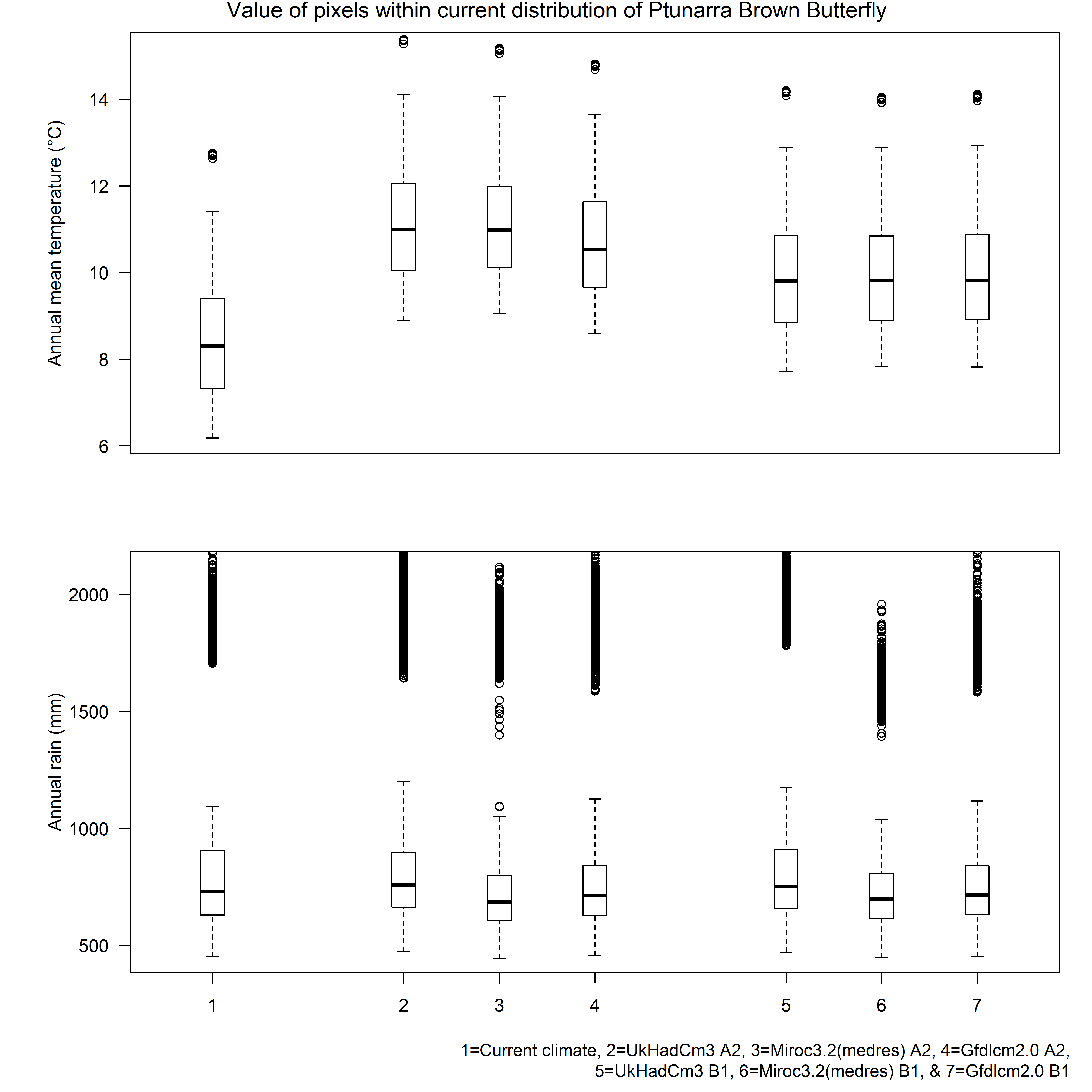

Supplement: Figure S4 — Differences in annual mean temperature and annual rain within the current distribution of the Ptunarra Brwon Butterfly. Where the box spans the interquartile range, the segment inside the box 45 shows the median and whiskers above and below the box show the locations of the minimum and maximum values; the circles represent outliers. (TIF) [file pone.0113749.s004.tif]

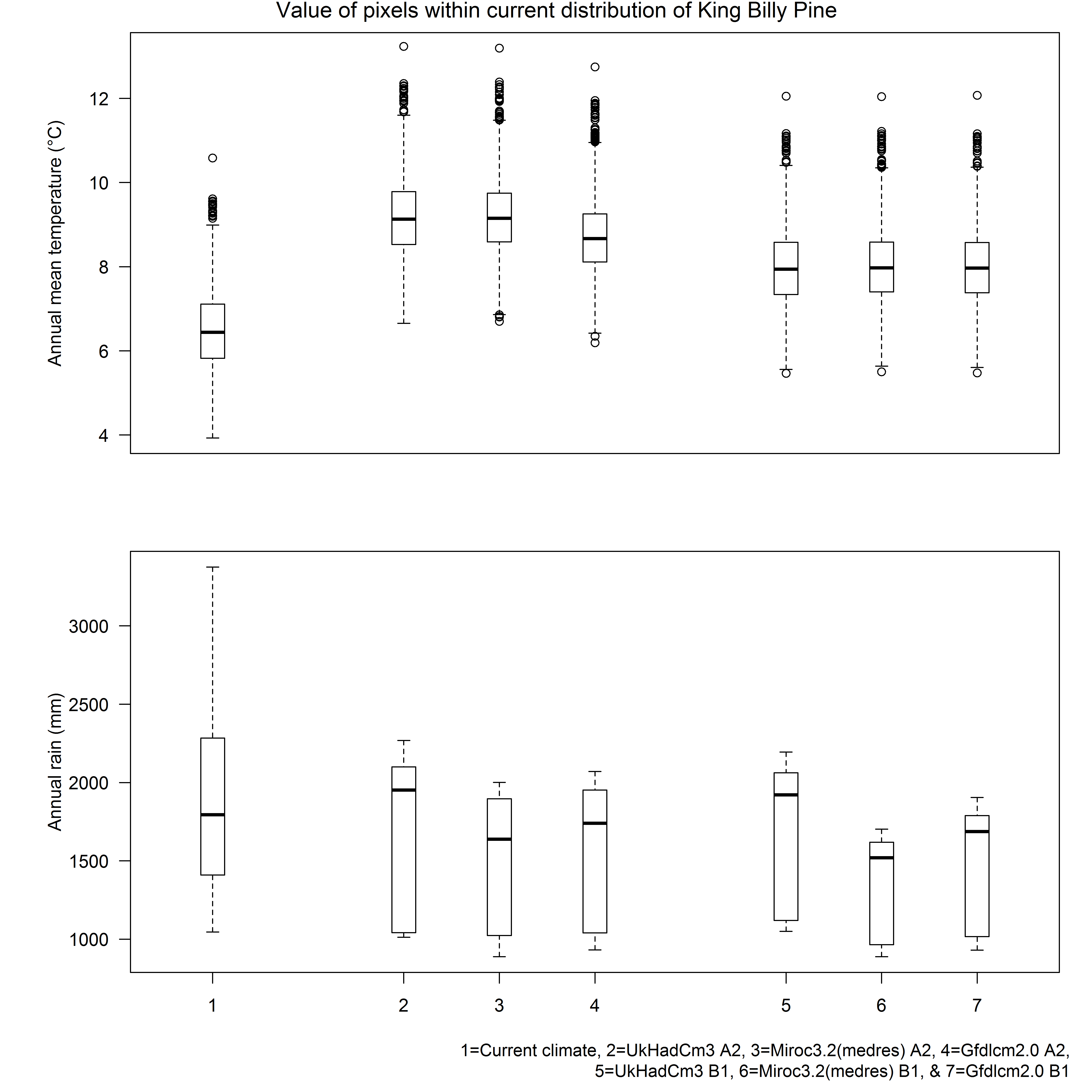

Supplement: Figure S5 — Differences in annual mean temperature and annual rain within the current distribution of the King Billy Pine. Where the box spans the interquartile range, the segment inside the box shows the median and whiskers above and below the box show the locations of the minimum and maximum values; the circles represent outliers. (TIF) [file pone.0113749.s005.tif]
