# Supplementary material for: Improving the Use of Species Distribution Models in Conservation Planning and Management under Climate Change
Source: PLoS One. 2014 Nov 24;9(11):e113749. doi: 10.1371/journal.pone.0113749 (PMC4242662; doi:10.1371/journal.pone.0113749)
Supplement: File S1 — Table A1, List of bioclimatic variables used in the SDM for Orexeinica ptunarra and Athrotaxis selaginoides. Subset of variable using a PCA is denoted by ‘PCA’ and the subset selection made by experts is denoted by ‘x’. The Orexeinica ptunarra's experts selected monthly variables. Table A2, The area predicted to be climatically suitable for the species based on the current and future climate projections. Pixels for the Ptunarra Brown Butterfly and the King Billy Pine that with a value0.5 or more were considered as ‘suitable’. The areas are shown in km2 for Models 1 to 3 based on the SRES scenario A2, and only for Model 3 based on SRES scenario B1. Maps were projected to 25 World Geodetic System 1984, Universal Transverse Mercator coordinate system Zone 55 South. (PDF) [file pone.0113749.s006.pdf]

Online Appendix

Commonly used climatic variables in SDMs

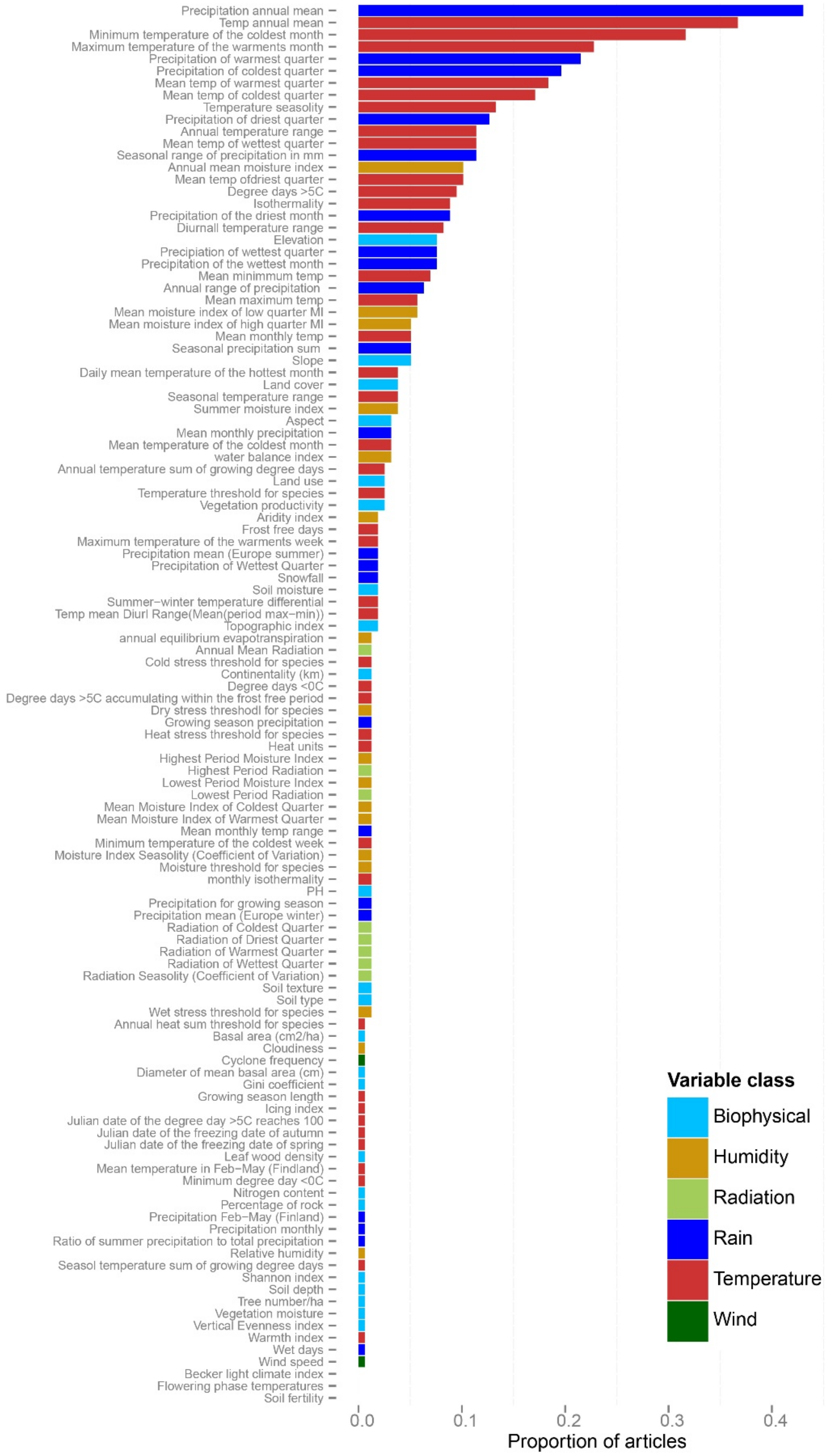

Figure A1. The proportion of variables used in the revised literature.

The bioclimatic variables

Table A1. List of bioclimatic variables used in the SDM for *Orexeinica ptunarra* and *Athrotaxis selaginoides*. Subset of variable using a PCA is denoted by ‘PCA’ and the subset selection made by experts is denoted by ‘x’. The *Orexeinica ptunarra*’s experts selected monthly variables.

	**Scientific name**	***Orexeinica ptunarra***	***Athrotaxis selaginoides***
	**Common name**	**Ptunarra Brown Butterfly**	**King Billy Pine**
**Bio ID**	**Bioclimatic parameters**		
bio1	Annual Mean Temperature		PCA
bio2	Mean Diurnal Range(Mean(period max-min))		
bio3*	Isothermality (bio2/bio7)		
bio4	Temperature Seasonality (Coefficient of Variation)	PCA	PCA
bio5	Max Temperature of Warmest Period	PCA	x
bio6	Min Temperature of Coldest Period		x
bio7	Temperature Annual Range (bio5-bio6)		PCA
bio8	Mean Temperature of Wettest Quarter		
bio9	Mean Temperature of Driest Quarter	PCA	

bio10	Mean Temperature of Warmest Quarter		x
bio11	Mean Temperature of Coldest Quarter		x
bio12	Annual Precipitation	PCA	x, PCA
bio13	Precipitation of Wettest Period		
bio14	Precipitation of Driest Period		x
bio15	Precipitation Seasonality(Coefficient of Variation)		
bio16	Precipitation of Wettest Quarter		
bio17	Precipitation of Driest Quarter		
bio18	Precipitation of Warmest Quarter		
bio19	Precipitation of Coldest Quarter		
bio20	Annual Mean Radiation		PCA
bio21	Highest Period Radiation		
bio22	Lowest Period Radiation		
bio23	Radiation Seasonality (Coefficient of Variation)		PCA
bio24	Radiation of Wettest Quarter		
bio25	Radiation of Driest Quarter		
bio26	Radiation of Warmest Quarter		
bio27	Radiation of Coldest Quarter		

bio28	Annual Mean Moisture Index	x, PCA
bio29	Highest Period Moisture Index	PCA
bio30	Lowest Period Moisture Index	x
bio31*	Moisture Index Seasonality (CV)	
bio32	Mean Moisture Index of Highest Quarter MI	
bio33	Mean Moisture Index of Lowest Quarter MI	x
bio34	Mean Moisture Index of Warmest Quarter	
bio35	Mean Moisture Index of Coldest Quarter	

** Bio3 (isothermality) can be interpreted as the evenness of temperature over the course of a year, or a quantification of how large the day-to-night temperature oscillation is in comparison to the summer-to-winter oscillation. A value of 100 would represent a site where the diurnal temperature range is equal to the annual temperature range.

*Bio31, the coefficient of variation of the moisture index was not used, because there was a large area in western Tasmania that could not be calculated due to standard deviation values of zero (No Data values were produced in this region by ANUCLIM, which resulted in MaxEnt maps which excluded this region).

The differences between the models

Table A2: The area predicted to be climatically suitable for the species based on the current and future climate projections. Pixels for the Ptunarra Brown Butterfly and the King Billy Pine that with a value0.5 or more were considered as 'suitable'. The areas are shown in km^2 for Models 1 to 3 based on the SRES scenario A2, and only for Model 3 based on SRES scenario B1. Maps were projected to World Geodetic System 1984, Universal Transverse Mercator coordinate system Zone 55 South.

	**Climate model**	**Model 1 A2**	**Model 2 A2**	**Model 3 A2**	**Model 3 B1**
**Ptunarra Brown Butterfly**	Current Climate	2705	3823	4676	
	UKHadC3.1 (2080)	209	566	421	1685
	Gfdlcm2.0 (2080)	988	613	1814	1402
	Miroc2.0 (medres) (2080)	853	224	2932	888
**King Billy Pine**	Current Climate	3246	3806	4167	
	UKHadC3.1 (2080)	100	154	90	797
	Gfdlcm2.0 (2080)	19	150	24	1437
	Miroc2.0 (medres) (2080)	7	134	1	1109

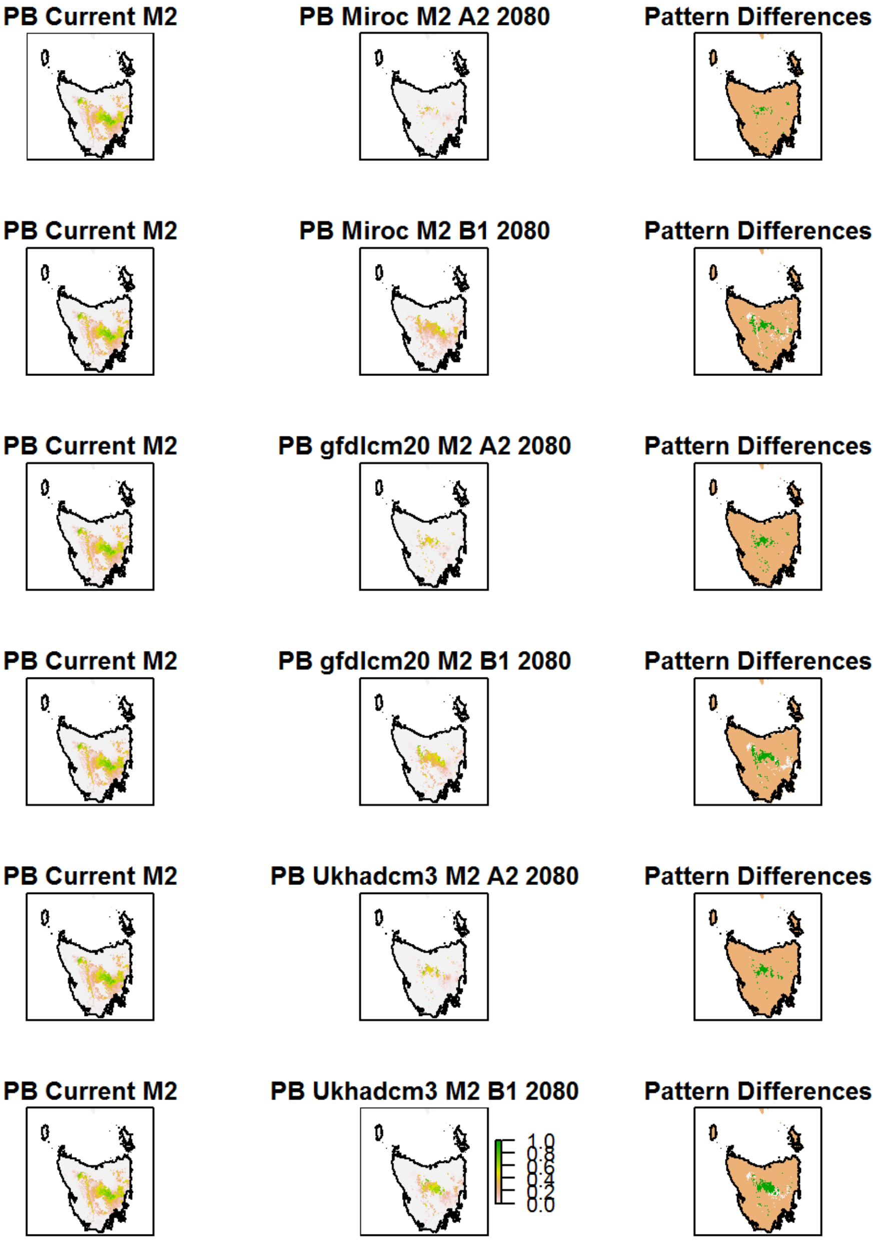

Key for Pattern difference maps: green = -ve, orange = ns, and white = +ve

Figure A2. Distribution maps of significant difference (SD) between the current prediction of suitable habitat for Ptunarra Brown (PB) based on baseline climate, and the future projections, based on Model 2 (M2, PCA selection of bioclimatic variables). The difference maps showed when the distribution predicted significantly more (+ve) or less suitable habitat (-ve) (SD $\geqslant$ 0.975 or SD $\leqslant$ 0.025, respectively) and where there was no significant (ns) difference between models.

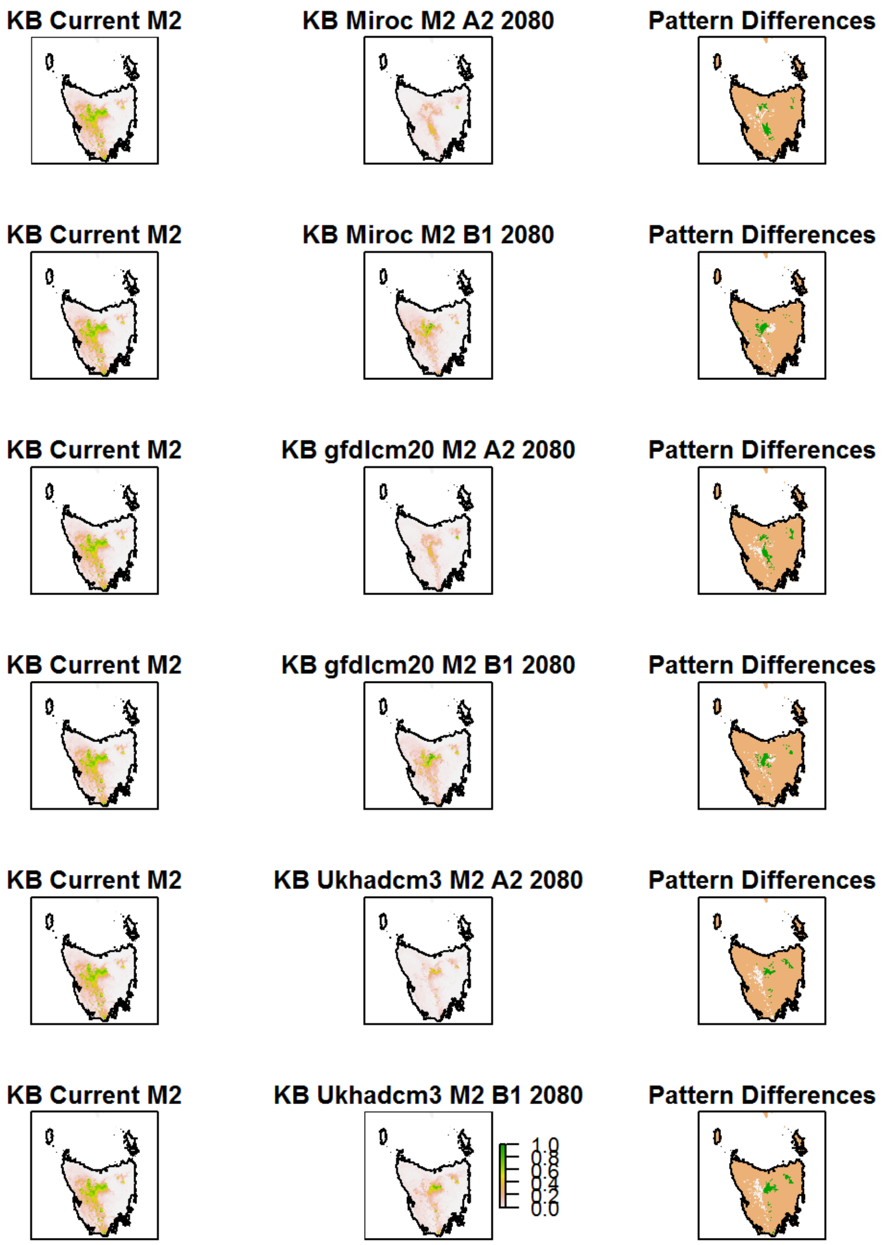

Key for Pattern difference maps: green = -ve, orange = ns, and white = +ve

Figure A3. Distribution maps of significant difference (SD) between the current prediction of suitable habitat for King Billy Pine (KB) based on baseline climate, and the future projections based on Model 2 (M2, PCA selection of bioclimatic variables). The difference maps showed when the

distribution predicted significantly more (+ve) or less suitable habitat (-ve) (SD ⩾ 0.975 or SD ⩽ 0.025, respectively) and where there was no significant (ns) difference between models.

The differences between the emissions scenarios

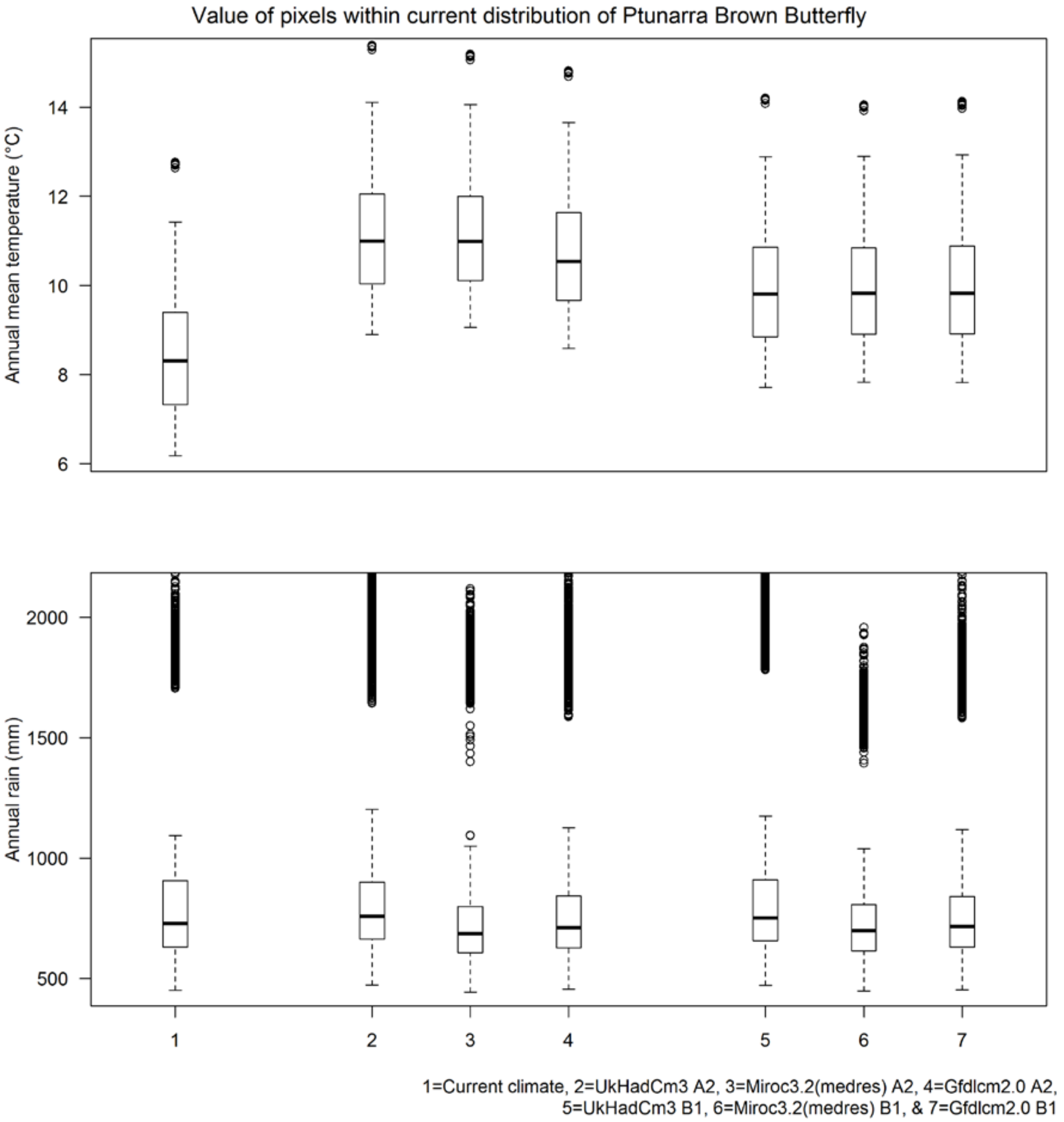

Figure A4. Differences in annual mean temperature and annual rain within the current distribution of the Ptunarra Brwon Butterfly. Where the box spans the interquartile range, the segment inside the box shows the median and whiskers above and below the box show the locations of the minimum and maximum values; the circles represent outliers.

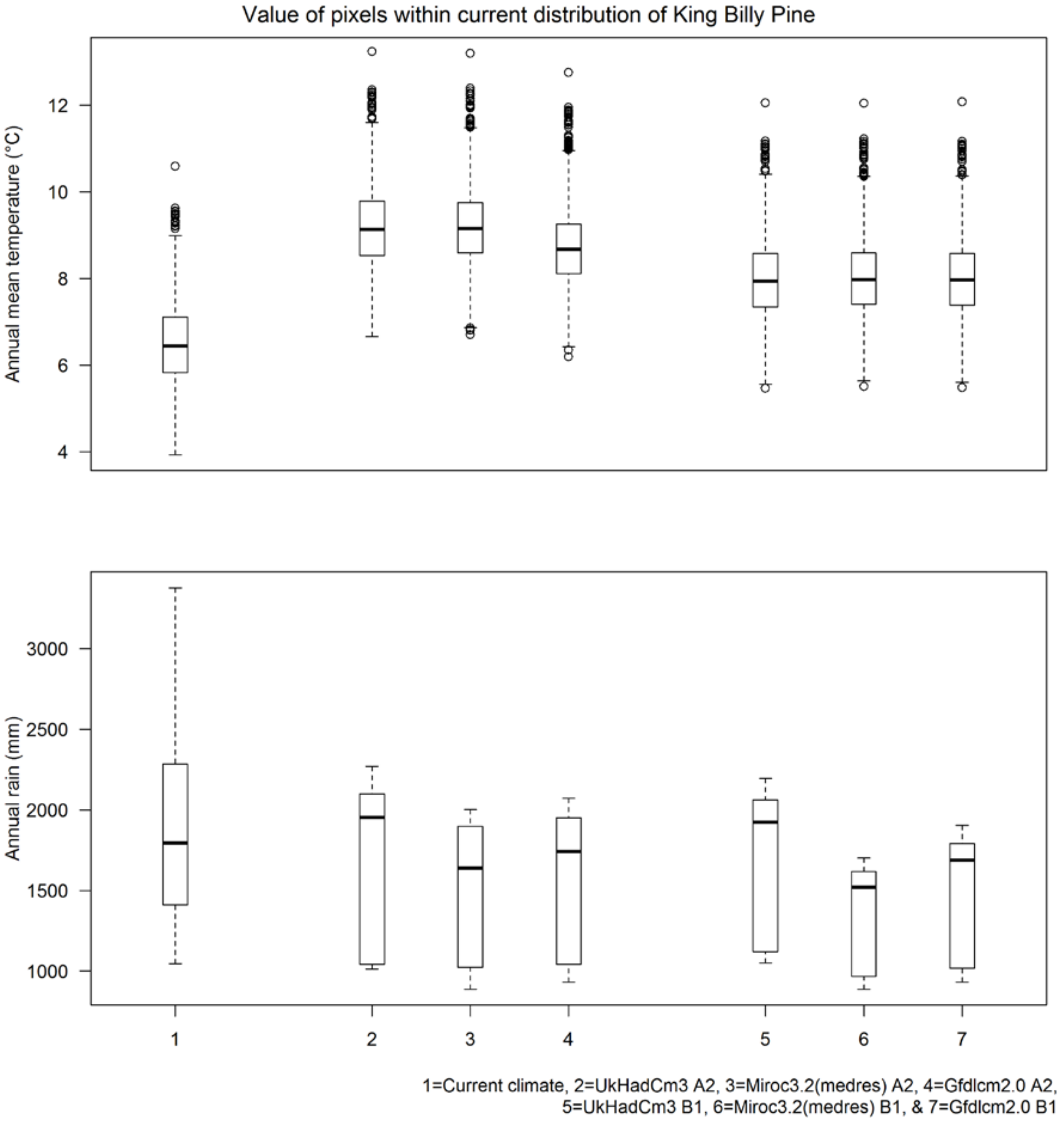

Figure A5. Differences in annual mean temperature and annual rain within the current distribution of the King Billy Pine. Where the box spans the interquartile range, the segment inside the box shows the median and whiskers above and below the box show the locations of the minimum and maximum values; the circles represent outliers.

Consulting the experts and conservation managers

Two meetings were organised at the Australian Department of Environment on the 5th September 2013 (33 Allara, St, Canberra ACT):

1. ***Carolyn Cameron*** of the Strategic Approaches Branch in the former Department of Sustainability, Environment, Water, Population and Communities (DSEWPaC) met with ***Ted Lefroy***, ***Bec Harris, Luciana Porfirio*** and ***Sonia Hugh*** to discuss the potential use by the branch of species distribution models that incorporate climate projections. The discussion focused on the way different distribution projections could be most effectively communicated and used by conservation planners and decision-makers undertaking strategic impact assessments and regional sustainability planning. The value of validating the climate change resilience of proposed landscape-scale offset sites was also discussed, as was the potential to apply the approach in other regions of Australia.
2. ***Ross Rowe*** facilitated a departmental information session on species distribution modelling under climate change for DSEWPaC officers responsible for sustainable regional development planning, environmental information and threatened species policy, listing and recovery. ***Bec Harris, Luciana Porfirio*** and ***Sonia Hugh*** discussed the key preliminary outcomes of their research and how these outcomes might be incorporated into recovery planning within the department. They emphasised the importance of three key decisions involved in species distribution modelling under future climate; the choice of global circulation model, the choice of emissions scenarios and the choice of environmental variables. Departmental officers attending included: ***Peter Wright, Peter Latch, Adam Cowell, Damian Wrigley, Dave Osborne*** and ***Simon Bennett.***

A key topic of the meetings was to answers the following question: “What is the most helpful way to present variability and uncertainties?” We presented different summary SDMs maps. The attendees agreed that a map showing agreement between models in addition to the current suitable climatic envelop was the most informative. Additional maps showing the range of uncertainty in predicted distributions were also helpful but less informative for decision making. Summary analysis of critical variables for different species also helpful. Useful application could be checking climate change resilience of potential offset areas identified in strategic assessments and sustainable regional development planning processes, listing, recovery and mapping of threatened species and communities.
